# Lidocaine vs. Other Local Anesthetics in the Development of Transient Neurologic Symptoms (TNS) Following Spinal Anesthesia: A Meta-Analysis of Randomized Controlled Trials

**DOI:** 10.3390/jcm9020493

**Published:** 2020-02-11

**Authors:** Chang-Hoon Koo, Hyun-Jung Shin, Sung-Hee Han, Jung-Hee Ryu

**Affiliations:** 1 Department of Anesthesiology & Pain Medicine, Seoul National University Bundang Hospital, Seongnam 13620, Korea; vollock9@gmail.com (C.-H.K.); medidoc@nate.com (H.-J.S.); noninvasive@hanmail.net (S.-H.H.); 2 Department of Anesthesiology & Pain Medicine, Seoul National University College of Medicine, Seoul 03080, Korea

**Keywords:** Lidocaine, Anesthesia, spinal, Postoperative Complications

## Abstract

The use of lidocaine in spinal anesthesia may increase the risk of transient neurological symptoms (TNS) according to previous meta-analyses. However, the previous meta-analyses lacked data on some other local anesthetics and thus, more evaluations are still needed to compare the effect of lidocaine on the development of TNS. The objective of this study was to compare the risk of TNS according to lidocaine versus other local anesthetics in patients undergoing spinal anesthesia. A total of 39 randomized controlled trials with 4733 patients were analyzed. The incidence of TNS was 10.8% in the lidocaine group and was 2.2% in the control groups (risk ratio (RR) 4.12, 95% confidence interval (CI) 3.13 to 5.43, *p* < 0.001). In subgroup analysis, lidocaine increased the incidence of TNS compared with other local anesthetics except mepivacaine, ropivacaine or sameridine. The risk of TNS was higher in the hyperbaric (*p* < 0.001) or isobaric lidocaine (*p* < 0.001) group compared with the control group, but there were no differences found between the two groups when hypobaric lidocaine was administered (*p* = 1.00). This study confirmed that lidocaine for spinal anesthesia still causes TNS more frequently than most other local anesthetics, especially when hyperbaric or isobaric lidocaine was used.

## 1. Introduction

Lidocaine is an attractive regional anesthetic for ambulatory surgery. It offers a rapid onset and fast recovery of both motor and sensory block [1]. However, when compared with other local anesthetics, the use of lidocaine in spinal anesthesia has been known to be associated with increased risk of transient neurological symptoms (TNS) [2,3], hindering its application in ambulatory spinal anesthesia. Other local anesthetics including mepivacaine, low-dose bupivacaine, procaine, articaine, levobupivacaine, ropivacaine and 2-chloroprocaine have been suggested as replacement drugs. 

TNS generally occurs in patients with single injection spinal anesthesia within the first 24 hours [2]. TNS consists of pain in the lower extremities without abnormalities in neurologic and radiologic examination [2]. In previous systematic reviews and meta-analyses [2,3], lidocaine has a significantly higher relative risk of developing TNS compared with most other local anesthetics, but the previous meta-analyses lacked data on ropivacaine, levobupivacaine, and chloropocaine; thus, more evaluations are still needed to confirm favorable results for these aforementioned local anesthetics. In the last decade, many randomized controlled trials (RCTs) comparing between the incidence of TNS after lidocaine and other local anesthetics have been conducted. More subsequent studies evaluating the effect of lidocaine on the risk of TNS have been published [2,3]. Furthermore, among them, many studies reported no patients suffering from TNS after spinal anesthesia with lidocaine [4,5,6,7,8,9,10,11,12,13,14,15,16,17,18]. Therefore, it is still controversial on the safety of lidocaine for spinal anesthesia during ambulatory surgery in terms of TNS. The objective of this systematic review and meta-analysis is to compare the incidence of TNS between lidocaine and other local anesthetics and to evaluate the frequency of TNS with various types of local anesthetics in adult surgical patients after spinal anesthesia.

## 2. Materials and Methods

### 2.1. Literature Search

This systematic review and meta-analysis was conducted according to the Preferred Items for Systematic Reviews and Meta Analyses (PRISMA) statements guideline [19]. A predefined protocol was registered in the International Prospective Register of Systematic Reviews (PROSPERO: CRD42019137819). RCTs comparing lidocaine versus other local anesthetics during spinal anesthesia were searched on the following databases: PubMed, EMBASE, CENTRAL, CINAHL, Scopus, Web of Science and KoreaMed. The final search was performed on March 31st, 2019. Search strategies were established with MeSH terms and keywords, including “spinal anesthesia”, “lidocaine”, or “lignocaine”. Each finding was combined with the Boolean operator, such as “AND”, “OR”. Detailed search strategies for each database were described in Table S1. The title, abstract, and authors of all retrieved articles were extracted and collected, regardless of the publication year, language or region.

### 2.2. Study Selection

C.-H.K. and H.-J.S. independently accessed the titles and abstracts of the articles to screen for relevant studies. Subsequently, full-texts of relevant articles were obtained via hand-search, library service or contacting the authors. C.-H.K. and J.-H.R. read the full text to select studies that were appropriate for this meta-analysis. The inclusion criteria were (1) randomized controlled trials, (2) surgical patients under spinal anesthesia, (3) lidocaine use for spinal anesthesia in at least in one group, and (4) use of other local anesthetic for spinal anesthesia in the control group. The exclusion criteria were: (1) abstract, protocol, conference poster or review; and (2) The study which did not report the incidence of TNS. S.-H.H. participated on selection if any disagreement existed.

### 2.3. Data Extraction

C.-H.K. and H.-J.S. independently investigated and collected the following data from final full-texts: author, publication year, language, sample size, type of surgery, type of anesthesia, patient’s position during surgery, needle type, characteristics of lidocaine (concentration, baricity, dose, and adjuvants), characteristics of local anesthetics used in the control group (type, concentration, baricity, dose, and adjuvants) and the incidence of TNS.

### 2.4. Risk of Bias Assessment

C.-H.K. and J.-H.R. independently assessed the risk of bias of the included studies using the Cochran Risk of Bias tool [20]. It consists of seven items: random sequence generation, allocation concealment, blinding of participants, blinding of outcome assessors, incomplete outcome data, selective reporting, and other biases. Each item was graded as low, unclear or high. S.-H.H. settled any disagreements between the aforementioned assessors.

### 2.5. Data Synthesis and Statistical Analysis

Data synthesis and meta-analysis were performed using Revman 5.3 software (Cochrane Collaboration, Oxford, UK) and R version 3.6.1 (R Foundation for Statistical Computing, Vienna, Austria). Since the incidence of TNS was dichotomous variable, the authors calculated the risk ratio (RR) as a pooled estimate. The inverse variance method and random effect models were employed. A continuity correction of 0.5 was applied to zero total events trials [21]. The findings were presented as a forest plot with 95% confidence intervals. A subgroup analysis was conducted according to the local anesthetics which were used in the control group. Additional subgroup analyses were carried out to investigate any relationship between the incidence of TNS and baricity/concentration. The heterogeneity among the studies was evaluated by I^2^ statistic. I^2^ could be interpreted in the following manner: 0% to 40% might not be important; 30% to 60% may represent moderate heterogeneity; 50% to 90% may represent substantial heterogeneity; and 75% to 100% considerable heterogeneity [22]. A publication bias was assessed by construction of a funnel plot and the linear regression test. Sensitivity analysis (leave one study out) was conducted to confirm the robustness of the results. 

## 3. Results

### 3.1. Descriptions of Trials

**Figure 1:**
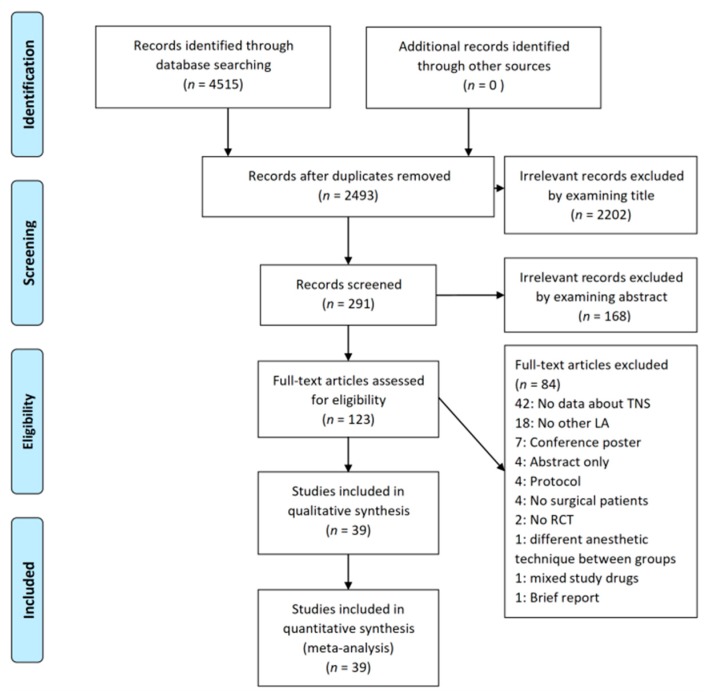
Flow diagram of the included and excluded studies. A total of 4515 articles were found during the literature search. Among them, 2493 articles were duplicated retrievals. A total of 2202 articles and 168 articles were obviously irrelevant studies. We excluded 84 articles due to various reasons. Finally, 39 articles were included in the final analysis. Abbreviations: TNS = transient neurological symptoms, LA = local anesthetics, RCT = randomized controlled trial

A total of 4515 articles were found on the initial database search. Among them, 2493 articles were removed due to duplication. Subsequently, 2202 articles and 168 articles were considered as irrelevant based on their title and abstract, respectively. The full-text of 123 articles were evaluated, and then, 84 articles were excluded due to the following reasons: no results about the incidence of TNS (*n* = 42); no other local anesthetics were used (*n* = 18); conference posters (*n* = 7); abstracts only (*n* = 4); protocols (*n* = 4); healthy subjects (*n* = 4); non-randomized studies of intervention (*n* = 2); different anesthetic techniques between groups (*n* = 1); mixed spinal anesthetics (*n* = 1); and a brief report (*n* = 1). Therefore, a total of 39 RCTs were included in the final analysis (Figure 1) [4,5,6,7,8,9,10,11,12,13,14,15,16,17,18,23,24,25,26,27,28,29,30,31,32,33,34,35,36,37,38,39,40,41,42,43,44,45,46]. 

All these RCTs compared lidocaine with other local anesthetics (bupivacaine, prilocaine, mepivacaine, levobupivacaine, chloroprocaine, ropivacaine, procaine, articaine, or sameridine) and reported the incidence of TNS. We found that 28 RCTs had two groups [4,5,6,7,8,9,10,11,12,14,15,18,23,26,27,29,30,34,35,36,37,38,39,40,41,42,44,46] of lidocaine more than other local anesthetics, while 11 RCTs had multiple groups [13,16,17,24,25,28,31,32,33,43,45]. Five out of 11 RCTs have more than two lidocaine groups [16,24,28,33,43]. According to the Cochrane guidelines [22], all lidocaine groups were pooled into a single group. In another five RCTs, more than two other local anesthetics were used for spinal anesthesia [17,25,31,32,45]. In the remaining RCT [13], three doses of sameridine were used for spinal anesthesia and the results of three sameridine groups were combined. Patients who received general anesthesia due to insufficient spinal block were excluded from the final analysis because it was unclear whether local anesthetics were administered in the cerebrospinal fluid. Details of each trial are summarized in Table 1. The 5% hyperbaric lidocaine was most used, followed by 2% isobaric. The dose of lidocaine used in each trial varied from 10 to 100mg. Specific details, including concentration, baricity, doses and adjuvants of study drugs, are summarized in Table 2.

### 3.2. Methodology Quality and Risk of Bias

**Figure 2:**
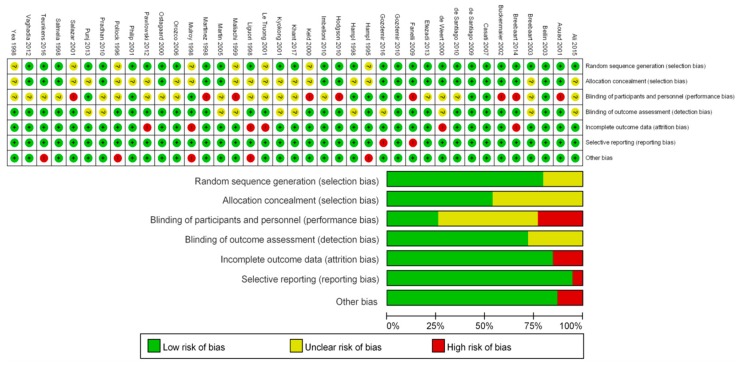
Risk of bias summary and graph using the Cochrane Risk of Bias tool [20]. The randomization and allocation concealment were well performed in most studies, but the method was not described in several studies. The performance bias was unclear or high in most studies, whereas the detection bias was low overall. The risk of attrition bias, reporting bias and other biases were low in most studies. Abbreviations: + = low risk of bias, ? = unclear risk of bias, - = high risk of bias.

The methodology quality and risk of bias are summarized in Figure 2. In each study, all patients were randomized to receive intrathecal lidocaine or other local anesthetics; however, the randomization method was unclear in eight studies. Twenty one studies maintained allocation concealment, but the other studies failed to describe it clearly. The risk of performance bias was high in 9 studies and unclear in 20 studies. It might be important for anesthesiologists to be aware of drugs for patient safety and sufficient block when performing spinal anesthesia. Unlike performance bias, the risk of detection bias was low overall. The risk of attrition bias, reporting bias, and other biases were low in more than 75% of the studies evaluated. Reasons for each risk of bias are shown in Table S2.

### 3.3. Outcome Synthesis

**Figure 3:**
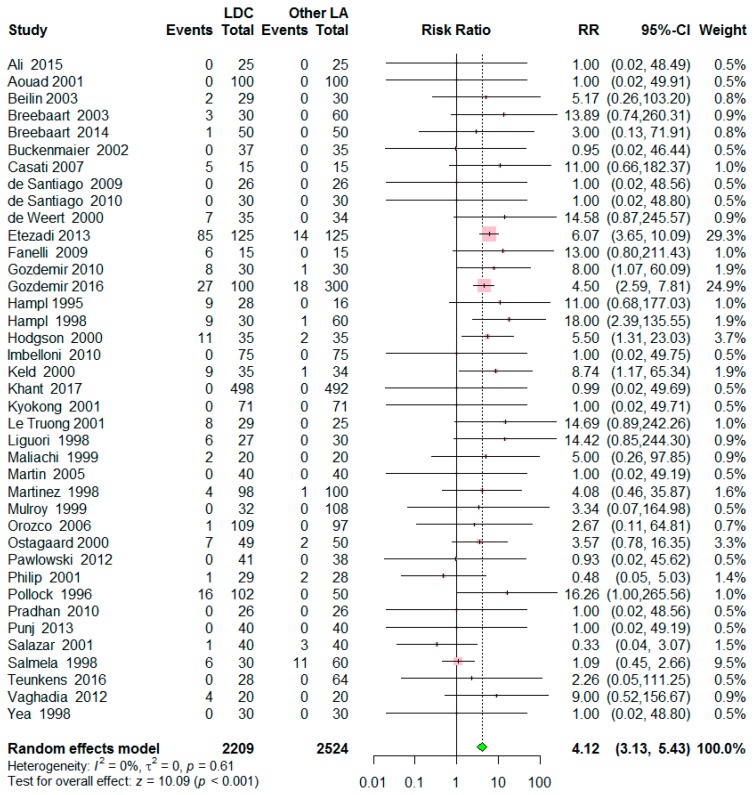
Forest plot for the risk of transient neurologic symptoms (TNS) after spinal anesthesia with lidocaine versus other local anesthetics. The incidence of TNS 10.8 % in the lidocaine group while 2.2% in the control group. The risk of TNS was significantly higher in the lidocaine group than in the control group (*p* < 0.001). Abbreviations: LDC = lidocaine, LA = local anesthetics, RR = risk ratio, CI = confidence interval.

A total of 39 studies included 4733 patients; 2209 patients were allocated to the lidocaine group and 2524 patients to the control group. The incidence of TNS was 10.8% (238/2209) in the lidocaine group and was 2.2% (56/2524) in the control group. The risk of TNS after spinal anesthesia was significantly higher in the lidocaine group than in the control group (Risk ratio (RR) = 4.12, 95% confidence interval (CI) = 3.13 to 5.43, *p* < 0.001), with a low level of heterogeneity (I^2^ = 0%, *p* = 0.61) (Figure 3). A symmetrical funnel plot and linear regression test showed insignificant results for publication bias (*p* = 0.206) (Figure S1). Sensitivity analysis revealed the robustness of the results (Table S3). Omitting one study [28] decreased the RR to 3.51, but still maintained the significance.

**Figure 4:**
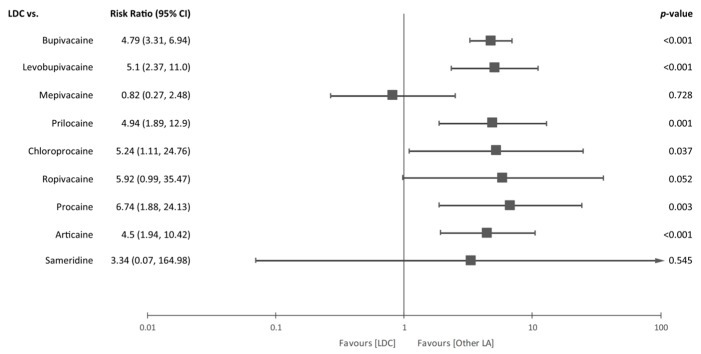
Forest plot for subgroup analysis. A subgroup analysis was conducted according to the local anesthetics which were used in the control group. The incidence of TNS was significantly higher in the lidocaine group than in the bupivacaine (*p* < 0.001), levobupivacaine (*p* < 0.001), prilocaine (*p* = 0.001), chloroprocaine (*p* = 0.037), procaine (*p* = 0.003) and articaine group (*p* < 0.001). However, there were no differences in the risk of TNS between the lidocaine group and mepivacaine (*p* =0.728), ropivacaine (*p* = 0.052) and sameridine group (*p* = 0.545). Abbreviations: LDC = lidocaine, LA = local anesthetics, CI = confidence interval.

In subgroup analysis as shown in Figure 4, lidocaine increased the incidence of TNS compared with most other local anesthetics, such as bupivacaine (RR = 4.79, 95% CI = 3.31 to 6.94, *p* < 0.001), levobupivacaine (RR = 5.1, 95% CI = 2.37 to 11.0, *p* < 0.001), prilocaine (RR = 4.94, 95% CI = 1.89 to 12.9, *p* = 0.001), chloroprocaine (RR = 5.24, 95% CI = 1.11 to 24.76, *p* = 0.037), procaine (RR = 6.74, 95% CI = 1.88 to 24.13, *p* = 0.003), and articaine (RR = 4.5, 95% CI = 1.94 to 10.42, *p* < 0.001). However, no significant difference was observed between the lidocaine group and the mepivacaine (RR = 0.82, 95% CI = 0.27 to 2.48, *p* = 0.728), ropivacaine (RR = 5.92, 95% CI = 0.99 to 35.47, *p* = 0.052) or sameridine group (RR = 3.34, 95% CI = 0.07 to 164.98, *p* = 0.545). A low level of heterogeneity was found in each subgroup analysis. 

**Figure 5:**
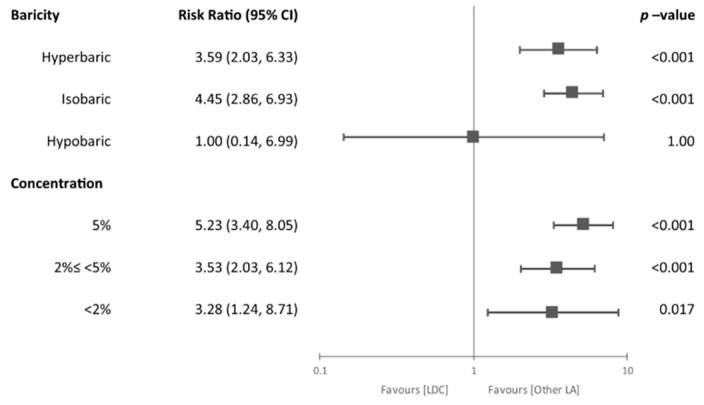
Forest plot for subgroup analysis. Additional subgroup analyses were conducted according to the baricity or concentration of the lidocaine. The risk of TNS was significantly higher in hyperbaric (*p* < 0.001), isobaric lidocaine group (*p* < 0.001) compared to the control group. In terms of concentration, lidocaine showed a higher incidence of TNS regardless of concentrations compared to the control group (*p* < 0.05). Abbreviations: LDC = lidocaine, LA = local anesthetics, CI = confidence interval.

The relationship between the incidence of TNS and baricity or concentration is shown in Figure 5. Sixteen studies used hyperbaric lidocaine [5,6,11,13,15,16,18,28,32,33,34,35,36,39,42,45], 12 studies used isobaric [14,17,23,24,25,26,27,29,30,31,41,44], and 4 studies used hypobaric lidocaine for spinal anesthesia [4,7,26,33]. One study had two treatment groups that administered hyperbaric or isobaric lidocaine [43]. Since 6 studies failed to report the baricity of lidocaine [10,12,37,38,40,46], those RCTs were excluded from subgroup analysis. The risk of TNS was higher in the lidocaine group compared with the control group when hyperbaric (RR = 3.59, 95% CI = 2.03 to 6.33, *p* < 0.001) or isobaric lidocaine (RR = 4.45, 95% CI = 2.86 to 6.93, *p* < 0.001) was used. However, there were no differences between the two groups with respect to the risk of TNS when hypobaric lidocaine was administered (RR = 1.00, 95% CI = 0.14 to 6.99, *p* = 1.00). As shown in Table 2, the concentration used in each study varied from 0.3% to 5%. Two studies have multiple groups with different concentration [16,43]. Most studies used 2% or 5% lidocaine and subgroups were categorized into 3 groups: (1) 5%, (2) 2%≤ <5%, and (3) <2%. The incidence of TNS was significantly higher in the lidocaine group compared with the control group in all categories (5%: RR = 5.23, 95% CI = 3.40 to 8.05, *p* < 0.001; 2%≤ <5%: RR = 3.53, 95% CI = 2.03 to 6.12, *p* < 0.001; <2%: RR = 3.28, 95% CI = 1.24 to 8.71, *p* = 0.017).

## 4. Discussion

The present meta-analysis confirmed that lidocaine used for spinal anesthesia still causes TNS more frequently than most other local anesthetics (bupivacaine, levobupivacaine, prilocaine, chloroprocaine, procaine, and articaine) except for mepivacaine, ropivacaine or sameridine. This is the first study that analyzed the role of baricity and concentration of lidocaine as potential risk factors for TNS, and the subgroup analysis showed that hyperbaric and isobaric lidocaine showed higher TNS rates than the others, and higher rates of TNS have been observed in all concentration categories of lidocaine.

The incidence of TNS of lidocaine was about 4 times higher than that of other local anesthetics in this study, which supported the result of the previous meta-analysis by Zaric et al. [2] which included 16 RCTs with a total of 1467 patients. Since then, many RCTs have been still conducted to compare the incidence of TNS between lidocaine and other local anesthetics. A recent meta-analysis including 24 studies with 2226 patients compared the risk of TNS by using direct and indirect comparison [3]. However, in this meta-analysis, more RCTs, including 39 studies with 4733 patients (more than twice) were analyzed and lidocaine was compared with more various local anesthetics. However, the risk ratio and prevalence of developing TNS of the current study was slightly lower than those of the previous studies; Zaric et al. (risk ratio 4.62, prevalence 14.2%) [2], Forget et al. (prevalence 18%) [3]. These difference can be explained in part by that many RCTs of the present meta-analysis reported no case of TNS in the lidocaine group [4,5,6,7,8,9,10,11,12,13,14,15,16,17,18], and a continuity correction of 0.5 was applied to these zero total events trials to prevent the overestimation of the risk of TNS [21]. Among other local anesthetics, chloroprocaine and mepivacaine have similar characteristics with lidocaine in terms of rapid onset time and short duration [47,48]. Subgroup analysis suggested that the incidence of TNS of chloroprocaine was lower than that of lidocaine. This finding significantly differs from the previous results [2,3]. However, the previous meta-analyses included only one or two RCTs comparing the effect of lidocaine and chloroprocaine. This study included four RCTs and no case of TNS in the chloroprocaine group was reported [17,24,26,46]. This would appear to indicate that chloroprocaine may be an attractive alternative to lidocaine for the short ambulatory surgery with fast onset and quick recovery time [48]. On the other hand, there was no difference in the incidence of TNS between lidocaine and mepivacaine, which was consistent with the result of the previous studies [2,3]. The idea that ropivacaine could decrease the development of TNS is still controversial. Although Zaric et al. [2] found that there was no difference in the risk of TNS between lidocaine and ropivacaine, Forget et al. [3] found that ropivacaine could decrease the risk of TNS than lidocaine. However, as expected, two studies included smaller number of studies and the latter study estimated pooled effect size by mostly indirect comparison. Surprisingly, the present study found more studies comparing the effect of lidocaine and ropivacaine, and estimated the pooled effect size by using a direct comparison.

Subgroup analysis suggested that no cases of TNS were found in the hypobaric lidocaine group whereas previous studies showed that the TNS of the lidocaine group occurred regardless of the baricity and concentration [49,50,51]. This result can be explained by low doses (10–20 mg) of hypobaric lidocaine group administered. Ben-David et al. [52] also reported that small doses of hypobaric lidocaine reduced the risk of TNS more than large doses of hypobaric lidocaine. However, this needs to be interpreted with caution since low doses of local anesthetics may be insufficient for adequate regional block [53]. Regarding the concentration of lidocaine, higher rates of TNS have been observed in all categories of concentration, which confirms the previous finding that altering the lidocaine concentration had no influence on the prevention of TNS [54]. 

This meta-analysis has a few limitations. First, various definitions of TNS were used in each study. Generally, TNS is defined as pain originating in the gluteal region and radiating to both lower extremities [2]. Some studies included considered TNS as only pain [4], while several other studies regarded TNS as pain and abnormal sensation (hypoesthesia or dysesthesia) [26,30]. Moreover, the anatomical regions (back, thigh, buttock or lower extremity), involving TNS, varied in each study. Furthermore, some studies did not specify details and/or a definition of TNS [35,47]. This variance with respect to TNS may have created bias, influencing the exact frequency of TNS. Second, specific types of surgery and position may be considered as risk factors of TNS, such as knee arthroscopy and lithotomy position. In the present study, various types of surgery and surgical position were included, and this may induce a bias in the results. Subgroup analysis according to the surgical position may provide a better overview. However, surgical position is heterogeneous and is not defined in some trials and the subgroup analysis according to the position, which may induce inaccurate results with bias. Third, only one study compared lidocaine to articaine with 134 patients, which may not be enough to conclude that the frequency of TNS with articaine is less than with lidocaine. Similarly, one RCT compared sameridine with lidocaine with 140 patients and there were no cases of TNS in the sameridine group.

## 5. Conclusions

In conclusion, the risk of developing TNS after spinal anesthesia with lidocaine was significantly higher than with bupivacaine, levobupivacaine, prilocaine, chloroprocaine, procaine or articaine. In addition, hyperbaric and isobaric lidocaine showed higher TNS rates than other lidocaines.

## Figures and Tables

**Table 1:** Baseline characteristics of the included randomized trials (*n* = 39).

Author	Year	Language	Anesthesia	Type of Operation	Number of Groups	Local Anesthetics (Control Group)	Needle	Position during Surgery	Follow-up Periods
Ali	2015	English	SA ^a^	Knee arthroscopy	2	Bupivacaine	25G Quincke	Supine	24,72,168 h
Aouad	2001	English	SA ^a^	Cesarean section	2	Bupivacaine	25G Whitacre	Supine	24,48,72 h
Beilin	2003	English	CSE ^b^	Cervical cerclage	2	Bupivacaine	25G Sprotte	Lithotomy	24 h
Breebaart	2003	English	SA ^a^	Knee arthroscopy	3	G1: Levobupivacaine G2: Ropivacaine	27G Whitacre	Supine	48 h
Breebaart	2014	English	SA ^a^	Knee arthroscopy	4	Chloroprocaine	27G Whitacre	Supine	168 h
Buckenmaier	2003	English	SA ^a^	Anorectal surgery	2	Ropivacaine	25G Pencan	Jackknife	24,48,72,168 h
Casati	2007	English	SA ^a^	Knee arthroscopy	2	Chloroprocaine	25G Whitacre	Not described	24, 168 h
de Santiago	2009	English	SA ^a^	Tubal sterilization	2	Levobupivacaine	27G Whitacre	Trendelenburg	168 h
de Santiago	2010	Spanish	SA ^a^	Anorectal surgery	2	Levobupivacaine	27G Whitacre	Jackknife	72, 168 h
de Weert	2000	English	SA ^a^	Short surgery of the lower body	2	Prilocaine	25G pencil-point	Supine	24 h
Etezadi	2013	English	SA ^a^	Varicocele, surgical fixation of lower extremities, transurethral resection of prostate, transurethral lithotripsy, herniorrhaphy	4	Bupivacaine	25G Sprotte or Quincke	Supine or lithotomy	8, 16, 24, 32, 40, 48, 72 h
Fanelli	2009	English	SA ^a^	Knee arthroscopy	2	Ropivacaine	25G Whitacre	Supine	24, 168 h
Gozdemir	2010	English	SA ^a^	Minor orthopedic, varicose vein, inguinal hernia, appendectomy	2	Levobupivacaine	25G Quincke	Supine	48, 168 h
Gozdemir	2016	English	SA ^a^	Minor orthopedic, cesarean section, varicose vein, inguinal hernia, appendectomy	4	G1: Levobupivacaine G2: Bupivacaine G3: Articaine	27G pencil-point	Not described	24,48,72 h
Hampl	1995	English	SA ^a^	Short gynecological procedure	3	Bupivacaine	25G pencil-point	Lithotomy	24 h
Hampl	1998	English	SA ^a^	Short gynecological procedure	3	G1: Prilocaine G2: Bupivacaine	25G pencil-point	Lithotomy	24 h
Hodgson	2000	English	SA ^a^	Knee arthroscopy	2	Procaine	24 or 25G pencil-point	Supine	72 h
Imbelloni	2010	English	SA ^a^	Anorectal surgery	2	Bupivacaine	27G Quincke	Jackknife	Until 30^th^ day
Keld	2000	English	SA ^a^	Inguinal hernia, femoral hernia, knee arthroscopy, removal of osteosynthetic material, fractures in the lower extremities, incision of infraumbilical abscess	2	Bupivacaine	25G pencil-point	Supine	24, 72 h
Khant	2017	English	SA ^a^	Urologic surgery	2	Bupivacaine	26G Quincke	Supine or lithotomy	Not described
Kyokong	2001	English	SA ^a^	Cesarean section	2	Bupivacaine	27G Quincke	Supine	24 h
Le Truong	2001	English	SA ^a^	General, gynecological, or other surgery	2	Procaine	27G Whitacre	Supine or lithotomy	48 h
Liguori	1998	English	SA ^a^	Knee arthroscopy	2	Mepivacaine	27G Whitacre	Supine	48 h
Maliachi	1999	Portuguese	SA ^a^	Femur surgery	2	Bupivacaine	22G, not described	Supine	24, 48, 72 h
Martin	2005	English	SA ^a^	Knee arthroscopy	2	Prilocaine	25G Whitacre	Not described	48h
Martinez	1998	English	SA ^a^	Orthopedic, urologic, gynecologic, vascular, general surgery	2	Prilocaine	25G pencil-point	Not described	72–120 h
Mulroy	1999	English	SA ^a^	Inguinal hernia	4	Sameridine	25G Whitacre	Supine	24 h
Orozco	2006	Spanish	SA ^a^	Surgery below the umbilicus	2	Bupivacaine	Not described	Not described	Not described
Ostgaard	2000	English	SA ^a^	Urology surgery	2	Prilocaine	25,26,27,29G Quincke	Supine or lithotomy	24 h
Pawlowski	2012	English	CSE ^b^	Anterior cruciate ligament repair	2	Mepivacaine	27G Pencan	Supine	24,48,72 h
Philip	2001	English	SA ^a^	Postpartum tubal ligation	2	Bupivacaine	25G Whitacre	Supine	24,48 h
Pollock	1996	English	SA ^a^	Knee arthroscopy or inguinal hernia	3	Bupivacaine	22 or 25G Quincke or Whitacre	Supine	72 h
Pradhan	2010	English	SA ^a^	Cesarean section	2	Bupivacaine	26G Quincke	Supine	Not described
Punj	2013	English	SA ^a^	pelvic surgery	4	Bupivacaine	24G Quincke	Supine	120 h
Salazar	2001	English	SA ^a^	Minor surgery of lower extremities	2	Mepivacaine	26 or 27G Quincke	Supine	24 h
Salmela	1998	English	SA ^a^	Urologic surgery, varicose vein, hemorrhoidectomy, hernia	3	G1: Mepivacaine G2: Bupivacaine	27G Quincke or Whitacre	Supine or lithotomy	24 h
Teunkens	2016	English	SA ^a^	Knee arthroscopy	3	G1: Chloroprocaine G2: Bupivacaine	27G Whitacre	Supine	24 h
Vaghadia	2012	English	SA ^a^	Transurethral resection of prostate	2	Chloroprocaine	25 or 27G Whitacre	Lithotomy	96–168 h
Yea	1998	Korean	SA ^a^	Surgery of lower body	2	Mepivacaine	25G Quincke	Supine	24 h

Abbreviations: ^a^ SA = spinal anesthesia; ^b^ CSE = combined spinal-epidural anesthesia.

**Table 2:** Concentration, baricity, doses and adjuvants of study drugs (*n* = 39).

Study	Sample Size		LDC ^a^				Control				
	LDC	Control	Concentration	Baricity	Dose	Added	Type	Concentration	Baricity	Dose	Added
Ali 2015	25	25	0.6%	Hypobaric	20 mg	FTN ^b^ 25 µg	Bupivacaine	0.375%	Hyperbaric	3 mg	FTN ^b^ 10 µg
Aouad 2001	100	100	5%	Hyperbaric	75 mg	-	Bupivacaine	0.75%	Hyperbaric	12 mg	-
Beilin 2003	29	30	1%	Isobaric	30 mg	FTN ^b^ 20 µg	Bupivacaine	0.175%	Hyperbaric	5.25 mg	FTN ^b^ 20 µg
Breebaart 2003	30	G1: 30 G2: 30	2%	Isobaric	60 mg	-	G1: Levobupivacaine G2: Ropivacaine	G1: 0.33% G2: 0.5%	G1:Isobaric G2:Isobaric	G1: 10 mg G2: 15 mg	-
Breebaart 2014	50	50	1.5%	Isobaric	60 mg	-	Chloroprocaine	1%	Isobaric	40 mg	-
Buckenmaier 2003	37	35	2.5%	Hyperbaric	25 mg	FTN ^b^ 20 µg	Ropivacaine	0.5%	Hyperbaric	4 mg	FTN ^b^ 20 µg
Casati 2007	15	15	1%	Isobaric	50 mg	-	Chloroprocaine	1%	Isobaric	50 mg	
de Santiago 2009	26	26	0.3%	Hypobaric	10 mg	FTN ^b^ 10 µg	Levobupivacaine	0.1%	Hypobaric	3 mg	FTN ^b^ 10 µg
de Santiago 2010	30	30	0.6%	Hypobaric	18 mg	FTN ^b^ 10 µg	Levobupivacaine	0.5%	Hypobaric	3 mg	FTN ^b^ 10 µg
de Weert 2000	35	34	2%	Isobaric	80 mg	-	Prilocaine	2%	Isobaric	80 mg	-
Etezadi 2013	125	125	5%	Hyperbaric	75–100 mg	-	Bupivacaine	0.5%	Isobaric	12.5–15 mg	-
Fanelli 2009	15	15	1%	Isobaric	50 mg	-	Ropivacaine	0.5%	Isobaric	10 mg	-
Gozdemir 2010	30	30	2%	Isobaric	80 mg	-	Levobupivacaine	0.5%	Isobaric	20 mg	-
Gozdemir 2016	100	G1: 100 G2: 100 G3: 100	2%	Isobaric	60 mg	-	G1: Levobupivacaine G2: Bupivacaine G3: Articaine	G1: 0.5% G2: 0.5% G3: 2%	G1: Isobaric G2: Isobaric G3: Isobaric	G1: 15 mg G2: 15 mg G3: 60 mg	-
Hampl 1995	G1: 15 G2: 13	16	G1:5% (7.5% dextrose) G2:5% (2.7% dextrose)	G1:Hyperbaric G2: Hyperbaric	G1:75 mg G2: 75 mg	-	Bupivacaine	0.5%	Hyperbaric	7.5 mg	-
Hampl 1998	30	G1: 30 G2: 30	2%	Hyperbaric	50 mg	-	G1: Prilocaine G2: Bupivacaine	G1: 2% G2: 0.5%	G1: Hyperbaric G2: Hyperbaric	G1: 50 mg G2:12.5 mg	-
Hodgson 2000	35	35	2.5%	Hyperbaric	50 mg	-	Procaine	5%	Hyperbaric	100 mg	
Imbelloni 2010	75	75	0.6%	Hypobaric	18 mg	-	Bupivacaine	0.15%	Hypobaric	4.5 mg	-
Keld 2000	35	34	5%	Hyperbaric	100 mg	-	Bupivacaine	0.5%	Hyperbaric	12.5 mg	-
Khant 2017	498	492	3.125%	Not described	25 mg	Butorphanol 0.3 mg	Bupivacaine	0.5%	Not described	5 mg	-
Kyokong 2001	71	71	5%	Hyperbaric	60 mg	MP^c^ 0.2 mg EPI^d^ 0.1 mg	Bupivacaine	0.5%	Hyperbaric	11 mg	MP^c^ 0.2 mg
Le Truong 2001	29	25	5%	Hyperbaric	100 mg	-	Procaine	5%	Isobaric	100 mg	-
Liguori 1998	27	30	2%	Not described	60 mg	-	Mepivacaine	1.5%	Not described	45 mg	-
Maliachi 1999	20	20	5%	Not described	1 mg/kg	-	Bupivacaine	0.5%	Not described	7–15 mg	-
Martin 2005	40	40	1.5%	Not described	45 mg	-	Prilocaine	1.5%	Not described	45 mg	-
Martinez 1998	98	100	5%	Hyperbaric	67.7 ± 8.7 mg	-	Prilocaine	5%	Hyperbaric	68.6±9.7 mg	-
Mulroy 1999	32	G1: 23 G2: 43 G3: 42	2.5%	Hyperbaric	100 mg	-	Sameridine	Not described	Isobaric	G1: 15 mg G2: 20 mg G3: 23 mg	-
Orozco 2006	109	97	5%	Not described	Not described	-	Bupivacaine	0.5%	Not described	Not described	-
Ostgaard 2000	49	50	2%	Isobaric	80 mg	-	Prilocaine	2%	Isobaric	80 mg	-
Pawlowski 2012	41	38	2%	Isobaric	80 mg	-	Mepivacaine	2%	Isobaric	80 mg	-
Philip 2001	29	28	5%	Hyperbaric	60–80 mg	-	Bupivacaine	0.75%	Hyperbaric	10.5–12 mg	-
Pollock 1996	G1:51 G2:51	50	G1: 5% G2: 2%	Hyperbaric or isobaric	60 or 75 mg	EPI ^d^ or none	Bupivacaine	0.75%	Hyperbaric	7.5 or 9 mg	-
Pradhan 2010	26	26	5%	Hyperbaric	75 mg	-	Bupivacaine	0.5%	Hyperbaric	12.5 mg	-
Punj 2013	G1:20 G2:20	G1: 20 G2: 20	G1: 5% G2: 2.5%	Hyperbaric	Not described	-	Bupivacaine	G1: 0.5% G2: 0.25%	Hyperbaric	G1: 10 mg G2: 5 mg	-
Salazar 2001	40	40	2%	Isobaric	40–60 mg	-	Mepivacaine	2%	Isobaric	40–60 mg	-
Salmela 1998	30	G1:30 G2: 30	2.5%	Hyperbaric	60–100 mg	-	G1: Mepivacaine G2: Bupivacaine	G1: 4% G2: 0.5%	G1: Hyperbaric G2: Hyperbaric	G1:40–80 mg G2:7.5–17 mg	-
Teunkens 2016	28	G1: 30 G2: 34	1%	Isobaric	40 mg	-	G1: Chloroprocaine G2: Bupivacaine	G1: 1% G2: 0.5%	G1: Isobaric G2: Isobaric	G1: 40 mg G2: 7.5 mg	
Vaghadia 2012	20	20	1.75%	Not described	35 mg	FTN ^b^ 12.5 µg	Chloroprocaine	1.77%	Isobaric	40 mg	FTN ^b^ 12.5 µg
Yea 1998	30	30	1.5%	Hyperbaric	75 mg	-	Mepivacaine	2%	Hyperbaric		-

Abbreviations: ^a^ LDC = Lidocaine; ^b^ FTN = Fentanyl; ^c^ MP = Morphine; ^d^ EPI = Epinephrine.

## Supplementary Materials

The following are available online at https://www.mdpi.com/2077-0383/9/2/493/s1, Figure S1: Funnel plot of comparison, lidocaine vs. other local anesthetics, Table S1: Search strategy for each database, Table S2: Details for judgement for each risk of bias, Table S3: Sensitivity analysis of comparison.
