# Supplementary material for: Lidocaine vs. Other Local Anesthetics in the Development of Transient Neurologic Symptoms (TNS) Following Spinal Anesthesia: A Meta-Analysis of Randomized Controlled Trials
Source: J Clin Med. 2020 Feb 11;9(2):493. doi: 10.3390/jcm9020493 (PMC7074456; doi:10.3390/jcm9020493)
Supplement: Supplementary file 1 [file jcm-09-00493-s001.zip › Table S1.docx]

Table S1. Search strategy for each database.

| Database | Order | Keywords | Results |
| --- | --- | --- | --- |
| PubMed | #1 | “Anesthesia, spinal”[MH] | 11817 |
|  | #2 | spinal[TIAB] AND anesth*[TIAB] | 18013 |
|  | #3 | spinal[TIAB] AND anaesth*[TIAB] | 6960 |
|  | #4 | #1 OR #2 OR #3 | 27644 |
|  | #5 | lidocaine[MH] | 23737 |
|  | #6 | lidocaine[TIAB] | 21046 |
|  | #7 | lignocaine[TIAB] | 2816 |
|  | #8 | #5 OR #6 OR #7 | 32045 |
|  | #9 | #4 AND #8 | 1635 |
|  | #10 | #9 AND HSSS(S) | 534 |
| EMBASE | #1 | ‘Spinal anesthesia’/exp | 23007 |
|  | #2 | ‘spinal anesth*’:ab,ti | 9573 |
|  | #3 | ‘spinal anaesth*’:ab,ti | 4964 |
|  | #4 | #1 OR #2 OR #3 | 25083 |
|  | #5 | lidocaine/exp | 71324 |
|  | #6 | lidocaine:ab,ti | 27452 |
|  | #7 | lignocaine:ab,ti | 3546 |
|  | #8 | #5 OR #6 OR #7 | 75018 |
|  | #9 | #4 AND #8 | 2930 |
|  | #10 | crossover procedure'/exp OR 'crossover procedure' OR 'double blind procedure'/exp OR 'double blind procedure' OR 'randomized controlled trial'/exp OR 'randomized controlled trial' OR 'single blind procedure'/exp OR 'single blind procedure' OR random* OR factorial* OR crossover* OR 'cross over' OR 'cross-over' OR placebo* OR (doubl* AND blind*) OR (singl* AND blind*) OR assign* OR allocat* OR volunteer* | 2376305 |
|  | #11 | #9 AND #10 | 760 |
|  | #12 | #11 AND [embase]/lim | 725 |
| CENTRAL | #1 | [mh “Anesthesia, Spinal”] | 2247 |
|  | #2 | spinal:ti,ab,kw AND anesth*:ti,ab,kw | 8366 |
|  | #3 | spinal:ti,ab,kw AND anaesth*:ti,ab,kw | 3122 |
|  | #4 | #1 OR #2 OR #3 | 9221 |
|  | #5 | [mh lidocaine] | 5364 |
|  | #6 | lidocaine:ti,ab,kw | 11063 |
|  | #7 | lignocaineti,ab,kw | 1529 |
|  | #8 | #5 OR #6 OR #7 | 11770 |
|  | #9 | #4 AND #8 | 864 |
|  | #10 | #9 in Trials | 860 |
| CINHAL | S1 | MH(Anesthesia, Spinal+) | 998 |
|  | S2 | TI(spinal anesth*) OR AB(spinal anesth*) | 410 |
|  | S3 | TI(spinal anaesth*) OR AB(spinal anaesth*) | 370 |
|  | S4 | S1 OR S2 OR S3 | 1238 |
|  | S5 | MH(lidocaine+) | 1471 |
|  | S6 | TI(lidocaine) OR AB(lidocaine) | 1207 |
|  | S7 | TI(lignocaine) OR AB(lignocaine) | 98 |
|  | S8 | S5 OR S6 OR S7 | 1946 |
|  | S9 | S4 AND S8 | 39 |
|  | S10 | (MH "Clinical Trials+") OR (PT Clinical trial) OR (TX clinic* n1 trial*) OR TX ( (singl* n1 blind*) OR (singl* n1 mask*) ) OR TX ( (doubl* n1 blind*) OR (doubl* n1 mask*) ) OR TX ( (tripl* n1 blind*) OR (tripl* n1 mask*) ) OR TX ( (trebl* n1 blind*) or (trebl* n1 mask*) ) OR TX randomi* control* trial* OR (MH "Random Assignment") OR TX random* allocat* OR TX placebo* OR (MH "Placebos") OR (MH "Quantitative Studies") OR TX allocat* random* | 632655 |
|  | S11 | S9 AND S10 | 24 |
| SCOPUS | #1 | INDEXTERMS(Anesthesia, Spinal) | 27631 |
|  | #2 | TITLE-ABS(spinal anesth*) | 26845 |
|  | #3 | TITLE-ABS(spinal anaesth*) | 9119 |
|  | #4 | #1 OR #2 OR #3 | 42656 |
|  | #5 | INDEXTERMS(lidocaine) | 68596 |
|  | #6 | TITLE-ABS(lidocaine) | 25231 |
|  | #7 | TITLE-ABS(lignocaine) | 3481 |
|  | #8 | #5 OR #6 OR #7 | 73904 |
|  | #9 | #4 AND #8 | 4379 |
|  | #10 | (INDEXTERMS(randomized controlled trial) OR INDEXTERMS(controlled clinical trial) OR TITLE-ABS(randomized) OR TITLE-ABS(placebo) OR INDEXTERMS(drug therapy) OR TITLE-ABS(randomly) OR TITLE-ABS(trial) OR TITLE-ABS(groups)) AND NOT (INDEXTERMS(animals) AND NOT INDEXTERMS(humans)) | 8620285 |
|  | #11 | #9 AND #10 | 1604 |
| Web of Science | #1 | TS=(spinal anesth*) | 19916 |
|  | #2 | TS=(spinall anaesth*) | 6362 |
|  | #3 | #1 OR #2 | 23658 |
|  | #4 | TS=(lidocaine or lingocaine) | 22984 |
|  | #5 | #3 AND #4 | 1574 |
|  | #6 | TS=(clinical trial* OR research design OR comparative stud* OR evaluation stud* OR controlled trial* OR follow-up stud* OR prospective stud* OR random* OR placebo* OR “single blind*” OR double blind*) | 4088470 |
|  | #7 | #5 AND #6 | 667 |
|  |  |  |  |
| KoreaMed | #1 | (spinal anesthesia) OR (spinal anesthetic) OR (spinal anesthetics) | 1287 |
|  | #2 | (lidocaine) OR (lingocaine) | 1081 |
|  | #3 | #1 And #2 | 101 |
