# Supplementary material for: Lidocaine vs. Other Local Anesthetics in the Development of Transient Neurologic Symptoms (TNS) Following Spinal Anesthesia: A Meta-Analysis of Randomized Controlled Trials
Source: J Clin Med. 2020 Feb 11;9(2):493. doi: 10.3390/jcm9020493 (PMC7074456; doi:10.3390/jcm9020493)
Supplement: Supplementary file 1 [file jcm-09-00493-s001.zip › Table S2.docx]

Table S2. Details for judgement for each risk of bias for randomized controlled studies.

| Study | Bias | Author’s judgement | Reason for judgement |
| --- | --- | --- | --- |
| Ali 2015 | Random sequence generation  (selection bias) | Low | Randomized by a computer generated random number table and by 1:1 ratio into two groups |
|  | Allocation concealment  (selection bias) | Unclear | Not described |
|  | Blinding  (performance) | Unclear | Not described |
|  | Blinding  (detection bias) | Unclear | Not described |
|  | Incomplete outcome data  (attrition bias) | Low | Outcomes were reported for all patients. |
|  | Selective reporting  (reporting bias) | Low | All pre-specified and expected outcomes are reported. |
|  | Other bias | Low | No other bias was detected. |
| Aouad 2001 | Random sequence generation  (selection bias) | Low | Patients were allocated randomly by sealed envelope |
|  | Allocation concealment  (selection bias) | Low | Patients were allocated randomly by sealed envelope |
|  | Blinding  (performance bias) | High | The anaesthesiologist who administered the spinal anesthetic and collected data on sensory and motor blockade was not blinded as to the study groups. |
|  | Blinding  (detection bias) | Low | All patients were interviewed on the first, second, and third postoperative days by an anesthesiologist who was unaware of the local anesthetic give. |
|  | Incomplete outcome data  (attrition bias) | Low | Outcomes were reported for all patients. |
|  | Selective reporting  (reporting bias) | Low | All pre-specified and expected outcomes are reported. |
|  | Other bias | Low | No other bias was detected. |
| Beilin 2003 | Random sequence generation  (selection bias) | Low | A computer-generated random number program was used to assign each subject |
|  | Allocation concealment  (selection bias) | Low | The results of the randomization were sealed in opaque envelopes |
|  | Blinding  (performance bias) | Low | The study medication was prepared by an anesthesiologist who was involved neither in the study nor in the clinical care of the patient. Neither the research assistant who performed all of the subsequent assessments nor the women were aware of study group assignment. |
|  | Blinding  (detection bias) | Low | A research assistant blinded to study group contacted each patient by telephone to inquire about any anesthetic complications, with a particular focus on TNS. |
|  | Incomplete outcome data  (attrition bias) | Low | Outcomes were reported for 59 patients. |
|  | Selective reporting  (reporting bias) | Low | All pre-specified and expected outcomes are reported. |
|  | Other bias | Low | No other bias was detected |
| Breebaart 2003 | Random sequence generation  (selection bias) | Low | By a computer-generated randomization sequence |
|  | Allocation concealment  (selection bias) | Unclear | Not described |
|  | Blinding  (performance bias) | Unclear | Not described |
|  | Blinding  (detection bias) | Unclear | Not described |
|  | Incomplete outcome data  (attrition bias) | Low | Outcomes were reported for all patients. |
|  | Selective reporting  (reporting bias) | Low | All pre-specified and expected outcomes are reported. |
|  | Other bias | Low | No other bias was detected. |
| Breebaart 2014 | Random sequence generation  (selection bias) | Low | Patients were randomized by sealed envelopes (computer generated allocation ) |
|  | Allocation concealment  (selection bias) | Low | Sealed envelopes |
|  | Blinding  (performance bias) | High | All people involved except the independent anaesthetist were blinded for the local anesthetic given. |
|  | Blinding  (detection bias) | Low | All people involved except the independent anaesthetist were blinded for the local anesthetic given. |
|  | Incomplete outcome data  (attrition bias) | High | After discharge, seven patients could not be reached by telephone for the 1 week follow-up. |
|  | Selective reporting  (reporting bias) | Low | All pre-specified and expected outcomes are reported. |
|  | Other bias | Low | No other bias was detected. |
| Buckenmaier 2002 | Random sequence generation  (selection bias) | Low | Simple randomization schedules were generated by computer by using a 1:1 proportion |
|  | Allocation concealment  (selection bias) | Low | The patient’s randomization was contained in an envelope |
|  | Blinding  (performance bias) | High | The attending anesthesiologist knew the spinal solutions before the anesthetic procedure. |
|  | Blinding  (detection bias) | Low | The research nurse (responsible for outcomes assessment), surgeon, and patient were unaware of the injected spinal solution. |
|  | Incomplete outcome data  (attrition bias) | Low | Five patients were excluded but outcomes were reported for all remained patients. |
|  | Selective reporting  (reporting bias) | Low | All pre-specified and expected outcomes are reported. |
|  | Other bias | Low | No other bias was detected. |
| Casati 2007 | Random sequence generation  (selection bias) | Low | Using a computer-generated sequence of numbers |
|  | Allocation concealment  (selection bias) | Low | Sealed envelopes |
|  | Blinding  (performance bias) | Low | The anesthesiologist performing the spinal injection, as well as the observers making assessments were blinded to patients grouping. |
|  | Blinding  (detection bias) | Low | The anesthesiologist performing the spinal injection, as well as the observers making assessments were blinded to patients grouping. |
|  | Incomplete outcome data  (attrition bias) | Low | Outcomes were reported for all patients. |
|  | Selective reporting  (reporting bias) | Low | All pre-specified and expected outcomes are reported. |
|  | Other bias | Low | No other bias was detected. |
| de Santiago 2009 | Random sequence generation  (selection bias) | Low | Patients were randomized into 2 groups by means of coded envelopes |
|  | Allocation concealment  (selection bias) | Low | Patients were randomized into 2 groups by means of coded envelopes |
|  | Blinding  (performance bias) | Low | The spinal solution was prepared by a nurse blinded to the study. All spinal anesthetics were administered by one of the authors who was familiar with the technique and was not involved in further patient evaluation. |
|  | Blinding  (detection bias) | Low | One week after surgery, a postoperative follow-up via telephone was done by a research assistant, blinded to the anesthetic solution used, who collected data about the occurrence to pain, TNSs, |
|  | Incomplete outcome data  (attrition bias) | Low | Outcomes were reported for all patients |
|  | Selective reporting  (reporting bias) | Low | All pre-specified and expected outcomes are reported. |
|  | Other bias | Low | No other bias detected. |
| de Santiago 2010 | Random sequence generation  (selection bias) | Low | The patients were randomly distributed into two group, according to a list of randomly generated numbers by a computer program (translated) |
|  | Allocation concealment  (selection bias) | Low | Placed in sealed envelopes (translated) |
|  | Blinding  (performance bias) | Unclear | There is no description. |
|  | Blinding  (detection bias) | Low | Another investigator, who also did not know the type of study solution that was administered to each patient, evaluated the progression of the blockade at 5,10,15 minutes and at the end of surgery (translated) |
|  | Incomplete outcome data  (attrition bias) | Low | Outcomes were reported for all patients |
|  | Selective reporting  (reporting bias) | Low | All pre-specified and expected outcomes are reported. |
|  | Other bias | Low | No other bias was detected. |
| de Weert 2000 | Random sequence generation  (selection bias) | Low | Patients were randomly allocated using sealed envelops |
|  | Allocation concealment  (selection bias) | Low | Sealed envelops |
|  | Blinding  (performance bias) | Unclear | Not described |
|  | Blinding  (detection bias) | Unclear | Not described |
|  | Incomplete outcome data  (attrition bias) | High | One patient in the prilocaine group could not be included because the data were incomplete. |
|  | Selective reporting  (reporting bias) | Low | All pre-specified and expected outcomes are reported. |
|  | Other bias | Low | No other bias was detected. |
| Fanelli 2009 | Random sequence generation  (selection bias) | Low | Using a computer-generated sequence of numbers for randomisation |
|  | Allocation concealment  (selection bias) | Low | Sealed envelopes for allocation concealment, |
|  | Blinding  (performance bias) | High | The anaesthesiologist performing the spinal block, who was aware of patient’s group allocation |
|  | Blinding  (detection bias) | Low | A blinded observer recorded progression and recovery from the spinal block, |
|  | Incomplete outcome data  (attrition bias) | Low | Outcomes were reported for all patients. |
|  | Selective reporting  (reporting bias) | High | One pre-defined outcome was not reported (dizziness). |
|  | Other bias | Low | No other bias was detected. |
| Etezadi 2013 | Random sequence generation  (selection bias) | Low | Randomization was achieved by a computer-generated block of numbers |
|  | Allocation concealment  (selection bias) | Low | Sealed envelope technique |
|  | Blinding  (performance bias) | Unclear | Not described |
|  | Blinding  (detection bias) | Low | The symptoms of TNS and any other complications were observed by a neurosurgeon that was blinded to surgical position of patients and the type of drugs and needles used for spinal anesthesia. |
|  | Incomplete outcome data  (attrition bias) | Low | Outcomes were reported for all patients. |
|  | Selective reporting  (reporting bias) | Low | All pre-specified and expected outcomes are reported. |
|  | Other bias | Low | No other bias was detected. |
| Gozdemir 2010 | Random sequence generation  (selection bias) | Low | Patients were assigned randomly by the authors via a sealed envelope method |
|  | Allocation concealment  (selection bias) | Low | Sealed envelope method |
|  | Blinding  (performance bias) | Low | Both drugs were prepared as 4ml doses and were drawn into syringes by an independent anaesthesia resident so that th anaesthetist performing the injection was unaware of which drug was being given. |
|  | Blinding  (detection bias) | Low | The investigators performing the interviews were unaware of which anaesthetic had been used. |
|  | Incomplete outcome data  (attrition bias) | Low | Outcomes were reported for all patients |
|  | Selective reporting  (reporting bias) | Low | All pre-specified and expected outcomes are reported. |
|  | Other bias | Low | No other bias was detected. |
| Gozdemir 2016 | Random sequence generation  (selection bias) | Low | Patients were assigned randomly by the authors via a sealed envelope method |
|  | Allocation concealment  (selection bias) | Low | Sealed envelope |
|  | Blinding  (performance bias) | Low | All drugs were prepared as 3ml doses and were drawn into syringes by an independent anesthesia resident so that thee anesthetist performing the injection was unaware of which drug has being given. |
|  | Blinding  (detection bias) | unclear | Not described |
|  | Incomplete outcome data  (attrition bias) | Low | Outcomes were reported for all patients |
|  | Selective reporting  (reporting bias) | High | Pre-defined outcome were not reported (headache, backache). |
|  | Other bias | Low | No other bias was detected. |
| Hampl 1995 | Random sequence generation  (selection bias) | Unclear | Patients were randomly assigned but the method was not described. |
|  | Allocation concealment  (selection bias) | Unclear | Not described |
|  | Blinding  (performance bias) | Low | The drugs were delivered by the pharmacy in blinded vials |
|  | Blinding  (detection bias) | Low | All patients were evaluated for TRI by oral interrogation by one anesthesiologist who was unware of details of the anesthesia procedure. |
|  | Incomplete outcome data  (attrition bias) | Low | One patient was excluded due to failed spinal anesthesia, but all other patients reported outcomes. |
|  | Selective reporting  (reporting bias) | Low | All pre-specified and expected outcomes are reported. |
|  | Other bias | High | The dosages of the study drugs were not equipotent as a consequence of the double-blind study design. |
| Hampl 1998 | Random sequence generation  (selection bias) | Low | Patients were assigned using a computer-generated randomization scheme |
|  | Allocation concealment  (selection bias) | Unclear | Not described |
|  | Blinding  (performance bias) | Low | The study solutions were provided in blinded vials |
|  | Blinding  (detection bias) | Unclear | Not described |
|  | Incomplete outcome data  (attrition bias) | Low | Outcomes were reported for all patients |
|  | Selective reporting  (reporting bias) | Low | All pre-specified and expected outcomes are reported. |
|  | Other bias | Low | No other bias was detected. |
| Hodgson 2000 | Random sequence generation  (selection bias) | Low | By computer-generated scheme |
|  | Allocation concealment  (selection bias) | Low | Blank, closed envelopes |
|  | Blinding  (performance bias) | High | Anesthesia team, which was not blinded to the spinal drug |
|  | Blinding  (detection bias) | Low | The patient remained blinded to the study drug. Patients were contacted by telephone approximately 3 days postoperatively by a single anesthesiologist blinded to the agent that the patient had received. |
|  | Incomplete outcome data  (attrition bias) | Low | Outcomes were reported for all patients |
|  | Selective reporting  (reporting bias) | Low | All pre-specified and expected outcomes are reported. |
|  | Other bias | Low | No other bias was detected. |
| Imbelloni 2010 | Random sequence generation  (selection bias) | Low | The randomized sequence was generated by a computer, |
|  | Allocation concealment  (selection bias) | Low | The preparation of coded envelopes |
|  | Blinding  (performance bias) | Unclear | Not described |
|  | Blinding  (detection bias) | Low | Proprioception and sensorial blockade were evaluated by another anesthesiologist, who was not aware of the groups. |
|  | Incomplete outcome data  (attrition bias) | Low | Outcomes were reported for all patients |
|  | Selective reporting  (reporting bias) | Low | All pre-specified and expected outcomes are reported. |
|  | Other bias | Low | No other bias was detected. |
| Keld 2000 | Random sequence generation  (selection bias) | Unclear | Patients were randomized, but the method was not described. |
|  | Allocation concealment  (selection bias) | Unclear | Not described |
|  | Blinding  (performance bias) | High | The anaesthesiologist used the local anaesthetic according to the random patient number and was thereofore not blinded to the anesthetic used. |
|  | Blinding  (detection bias) | Low | The patient was contacted on the first and third postoperative days by a different anaesthesiologist, who was blinded to the anaesthetic used and was only informed of the patients’ data and telephone number. |
|  | Incomplete outcome data  (attrition bias) | Low | One patient was excluded due to failed spinal anesthesia, but all other patients reported outcomes. |
|  | Selective reporting  (reporting bias) | Low | All pre-specified and expected outcomes are reported. |
|  | Other bias | Low | No other bias was detected. |
| Khant 2017 | Random sequence generation  (selection bias) | Low | Randomization was carried out using computer-generated simple random tables. |
|  | Allocation concealment  (selection bias) | Unclear | Not described |
|  | Blinding  (performance bias) | Unclear | Not described |
|  | Blinding  (detection bias) | Unclear | Not described |
|  | Incomplete outcome data  (attrition bias) | Low | Outcomes were reported for all patients |
|  | Selective reporting  (reporting bias) | Low | All pre-specified and expected outcomes are reported. |
|  | Other bias | Low | No other bias was detected. |
| Kyokong 2001 | Random sequence generation  (selection bias) | Low | All patients were randomized into two groups by random number table |
|  | Allocation concealment  (selection bias) | Unclear | Not described |
|  | Blinding  (performance bias) | Unclear | Not described |
|  | Blinding  (detection bias) | Unclear | Not described |
|  | Incomplete outcome data  (attrition bias) | Low | Outcomes were reported for all patients |
|  | Selective reporting  (reporting bias) | Low | All pre-specified and expected outcomes are reported. |
|  | Other bias | Low | No other bias was detected. |
| Le Truong 2001 | Random sequence generation  (selection bias) | Unclear | Patients were randomized, but the method was not described. |
|  | Allocation concealment  (selection bias) | Unclear | Not described |
|  | Blinding  (performance bias) | Unclear | Not described |
|  | Blinding  (detection bias) | Low | A blinded observer noted |
|  | Incomplete outcome data  (attrition bias) | High | Five patients were excluded in group P, and one patients were excluded in group L |
|  | Selective reporting  (reporting bias) | Low | All pre-specified and expected outcomes are reported. |
|  | Other bias | Low | No other bias was detected. |
| Liguori 1998 | Random sequence generation  (selection bias) | Low | Random number table |
|  | Allocation concealment  (selection bias) | Unclear | Not described |
|  | Blinding  (performance bias) | Unclear | Not described |
|  | Blinding  (detection bias) | Low | An investigator blinded to the local anesthetic assessed |
|  | Incomplete outcome data  (attrition bias) | High | Three patients in lidocaine group were excluded due to failed spinal anesthesia. |
|  | Selective reporting  (reporting bias) | Low | Not described |
|  | Other bias | High | Power analysis required 75 patients in each group, but because of concerns regarding the incidence of TNS with mepivacaine, the authors decided to do an interim analysis after 60 patients. |
| Maliachi 1999 | Random sequence generation  (selection bias) | Unclear | The composition of group was random, but the method was not described |
|  | Allocation concealment  (selection bias) | Unclear | Not described |
|  | Blinding  (performance bias) | High | The composition of the groups was only known by the anaesthesiologist in charge of the procedure |
|  | Blinding  (detection bias) | Unclear | Not described |
|  | Incomplete outcome data  (attrition bias) | Low | Outcomes were reported for all patients |
|  | Selective reporting  (reporting bias) | Low | All pre-specified and expected outcomes are reported. |
|  | Other bias | Low | No other bias was detected. |
| Martin 2005 | Random sequence generation  (selection bias) | Low | By means of a sealed, numbered envelope |
|  | Allocation concealment  (selection bias) | Low | A sealed, numbered envelope |
|  | Blinding  (performance bias) | Unclear | Not described |
|  | Blinding  (detection bias) | Unclear | Not described |
|  | Incomplete outcome data  (attrition bias) | Low | Outcomes were reported for all patients |
|  | Selective reporting  (reporting bias) | Low | All pre-specified and expected outcomes are reported. |
|  | Other bias | Low | No other bias was detected. |
| Martinez 1998 | Random sequence generation  (selection bias) | Low | A computer-generated list by simple random sampling |
|  | Allocation concealment  (selection bias) | Low | Patients were numbered consecutively by a blinded observer and allocated to one of the anesthetic solution. |
|  | Blinding  (performance bias) | High | The anesthetist who administered the spinal anesthesia was not blinded to the study groups. |
|  | Blinding  (detection bias) | Low | by an anesthesiologist who was unaware of the drug given or details of the anesthetic technique. |
|  | Incomplete outcome data  (attrition bias) | Low | Two patients were excluded due to failed spinal anesthesia, but outcomes were reported for all remained patients |
|  | Selective reporting  (reporting bias) | Low | All pre-specified and expected outcomes are reported. |
|  | Other bias | Low | No other bias was detected. |
| Mulroy 1998 | Random sequence generation  (selection bias) | Unclear | Randomized, but the method was not described |
|  | Allocation concealment  (selection bias) | Unclear | Not described |
|  | Blinding  (performance bias) | Low | Anesthetics were administered by a physician blinded to the randomization |
|  | Blinding  (detection bias) | Low | Postoperative care and data collection were performed by personnel blinded to the randomization |
|  | Incomplete outcome data  (attrition bias) | High | The sameridine 15mg dose group was excluded from further accrual after 35 subjects were enrolled because of the occurrence of eight failures |
|  | Selective reporting  (reporting bias) | Low | All pre-specified and expected outcomes are reported. |
|  | Other bias | High | As a multicenter study, there were variations in the treatment |
| Orozco 2006 | Random sequence generation  (selection bias) | Low | By random number table (translated) |
|  | Allocation concealment  (selection bias) | Unclear | Not described |
|  | Blinding  (performance bias) | Unclear | Not described |
|  | Blinding  (detection bias) | Low | Investigator did not know the medication used in each patient (translated) |
|  | Incomplete outcome data  (attrition bias) | Low | 5 patients were excluded, but outcomes were reported for all remained patients |
|  | Selective reporting  (reporting bias) | Low | All pre-specified and expected outcomes are reported. |
|  | Other bias | Low | No other bias was detected. |
| Ostgaard 2000 | Random sequence generation  (selection bias) | Low | Patients were randomized in the morning using sealed envelopes |
|  | Allocation concealment  (selection bias) | Low | Sealed envelopes |
|  | Blinding  (performance bias) | Unclear | Not described |
|  | Blinding  (detection bias) | Low | Patients were interviewed by an anaesthesiologist unaware of the local anaesthetic given |
|  | Incomplete outcome data  (attrition bias) | Low | Outcomes were reported for all patients |
|  | Selective reporting  (reporting bias) | Low | All pre-specified and expected outcomes are reported. |
|  | Other bias | Low | No other bias was detected. |
| Pawlowski 2012 | Random sequence generation  (selection bias) | Low | Computer-generated random sequence |
|  | Allocation concealment  (selection bias) | Unclear | Not described |
|  | Blinding  (performance bias) | Unclear | Not described |
|  | Blinding  (detection bias) | Low | Study-blinded observer assessed |
|  | Incomplete outcome data  (attrition bias) | High | Two patients were lost to late and long-term follow-up |
|  | Selective reporting  (reporting bias) | Low | All pre-specified and expected outcomes are reported. |
|  | Other bias | Low | No other bias was detected. |
| Philip 2001 | Random sequence generation  (selection bias) | Low | Double-blinded manner using a computer-generated randomization scheme |
|  | Allocation concealment  (selection bias) | Unclear | Not described |
|  | Blinding  (performance bias) | Low | The local anesthetic solutions were prepared in blinded vials |
|  | Blinding  (detection bias) | Low | Patients were interviewed by a research nurse who was blinded to the entire anesthetic. |
|  | Incomplete outcome data  (attrition bias) | Low | One patient was excluded due to failed spinal anesthesia, but outcomes were reported for all remained patients |
|  | Selective reporting  (reporting bias) | Low | All pre-specified and expected outcomes are reported. |
|  | Other bias | Low | No other bias was detected. |
| Pollock 1996 | Random sequence generation  (selection bias) | Unclear | Randomized, but the method was not described |
|  | Allocation concealment  (selection bias) | Unclear | Not described |
|  | Blinding  (performance bias) | Unclear | Not described |
|  | Blinding  (detection bias) | Low | Patients completed a telephone interview with a blinded investigator |
|  | Incomplete outcome data  (attrition bias) | Low | Seven patients were excluded due to insufficient spinal anesthesia, but outcomes were reported for all remained patients |
|  | Selective reporting  (reporting bias) | Low | All pre-specified and expected outcomes are reported. |
|  | Other bias | High | No attempt was made to equalize the numbers of patients having the two different types of surgery. |
| Pradhan 2010 | Random sequence generation  (selection bias) | Low | The patients were randomly divided into two groups according to the group in closed envelope |
|  | Allocation concealment  (selection bias) | Low | Closed envelope |
|  | Blinding  (performance bias) | Unclear | Not described |
|  | Blinding  (detection bias) | Unclear | Not described |
|  | Incomplete outcome data  (attrition bias) | Low | Outcomes were reported for all patients |
|  | Selective reporting  (reporting bias) | Low | All pre-specified and expected outcomes are reported. |
|  | Other bias | Low | No other bias was detected. |
| Punj 2013 | Random sequence generation  (selection bias) | Low | By a computer generated random number table |
|  | Allocation concealment  (selection bias) | Unclear | Not described |
|  | Blinding  (performance bias) | Low | The anaesthesiologist performing the spinal anesthetic was blinded to the solution being injected |
|  | Blinding  (detection bias) | Unclear | Not described |
|  | Incomplete outcome data  (attrition bias) | Low | Outcomes were reported for all patients |
|  | Selective reporting  (reporting bias) | Low | All pre-specified and expected outcomes are reported. |
|  | Other bias | Low | No other bias was detected. |
| Salazar 2001 | Random sequence generation  (selection bias) | Unclear | Randomized, but the method was not described |
|  | Allocation concealment  (selection bias) | Unclear | Not described |
|  | Blinding  (performance bias) | High | The anaesthesiologist who administered the spinal anaesthesia and recorded all the anaesthetic and intraoperative data was not blinded to the study groups |
|  | Blinding  (detection bias) | Low | The anaesthesiologist assessing the postoperative incidence of TNS was blinded to the group allocation of the patient |
|  | Incomplete outcome data  (attrition bias) | Low | Outcomes were reported for all patients |
|  | Selective reporting  (reporting bias) | Low | All pre-specified and expected outcomes are reported. |
|  | Other bias | Low | No other bias was detected. |
| Salmela 1998 | Random sequence generation  (selection bias) | Low | Patients were randomized using sealed envelopes |
|  | Allocation concealment  (selection bias) | Low | Sealed envelopes |
|  | Blinding  (performance bias) | Unclear | Not described |
|  | Blinding  (detection bias) | Low | The patients were interviewed using a standardized questionnaire on the first postoperative day by an anesthesiologist who did not know which spinal anesthetic agent had been used |
|  | Incomplete outcome data  (attrition bias) | Low | Outcomes were reported for all patients |
|  | Selective reporting  (reporting bias) | Low | All pre-specified and expected outcomes are reported. |
|  | Other bias | Low | No other bias was detected. |
| Teunkens 2016 | Random sequence generation  (selection bias) | Low | Using a computer-generated random table |
|  | Allocation concealment  (selection bias) | Low | Allocation concealment was ensured by enclosing assignments in sealed, opaque, sequentially numbered envelopes. |
|  | Blinding  (performance bias) | Unclear | Not described |
|  | Blinding  (detection bias) | Low | All data were collected by the study nurse of the department who was blinded to the treatment |
|  | Incomplete outcome data  (attrition bias) | Low | Six patients were excluded due to failed spinal anesthesia, but outcomes were reported for all remained patients |
|  | Selective reporting  (reporting bias) | Low | All pre-specified and expected outcomes are reported. |
|  | Other bias | High | Routine use of paracetamol and/or ketorolac might have probably masked possible symptoms of TNS in our patients. |
| Vaghadia 2012 | Random sequence generation  (selection bias) | Low | Compture-generated randomization |
|  | Allocation concealment  (selection bias) | Low | A sealed coded envelope technique |
|  | Blinding  (performance bias) | Unclear | Not described |
|  | Blinding  (detection bias) | Low | An investigator who was blinded to the study drug assessed the recovery of sensory and motor block until discharge criteria were achieved. A telephone interview was conducted by a blinded investigator to inquire about the incidence of following |
|  | Incomplete outcome data  (attrition bias) | Low | There were no dropouts or case failures |
|  | Selective reporting  (reporting bias) | Low | All pre-specified and expected outcomes are reported. |
|  | Other bias | Low | No other bias was detected. |
| Yea 1998 | Random sequence generation  (selection bias) | Unclear | Randomized, but the method was not described |
|  | Allocation concealment  (selection bias) | Unclear | Not described |
|  | Blinding  (performance bias) | Unclear | Not described |
|  | Blinding  (detection bias) | Low | A blinded observer |
|  | Incomplete outcome data  (attrition bias) | Low | Outcomes were reported for all patients |
|  | Selective reporting  (reporting bias) | Low | All pre-specified and expected outcomes are reported. |
|  | Other bias | Low | No other bias was detected. |
