# Supplementary figures and images for: Lidocaine vs. Other Local Anesthetics in the Development of Transient Neurologic Symptoms (TNS) Following Spinal Anesthesia: A Meta-Analysis of Randomized Controlled Trials
Source: J Clin Med. 2020 Feb 11;9(2):493. doi: 10.3390/jcm9020493 (PMC7074456; doi:10.3390/jcm9020493)

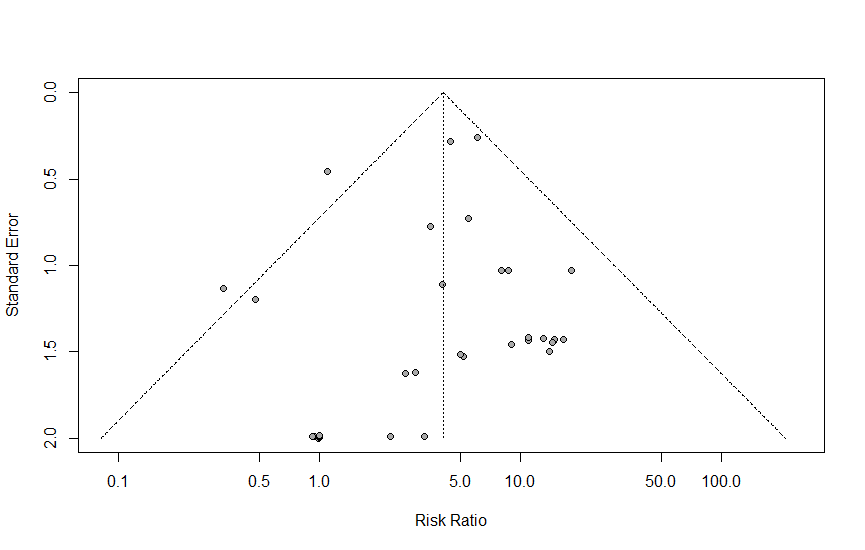

Supplement: Supplementary file 1 [file jcm-09-00493-s001.zip › Figure S1.tiff]
